# Neutrophil-to-lymphocyte ratio as a predictor of all-cause and cardiovascular mortality in coronary heart disease and hypertensive patients: a retrospective cohort study

**DOI:** 10.3389/fendo.2024.1442165

**Published:** 2024-08-21

**Authors:** Songhong Song, Liwen Chen, Rong Yu, Jinxiu Zhu

**Affiliations:** ^1^ Department of Preventive Medicine, Shantou University Medical College, Shantou, China; ^2^ Institute of Clinical Electrocardiology, First Affiliated Hospital of Shantou University Medical College, Shantou, China; ^3^ Longgang Maternity and Child Institute of Shantou University Medical College (Longgang District Maternity & Child Healthcare Hospital of Shenzhen City), Shenzhen, China

**Keywords:** coronary heart disease, hypertension, neutrophil lymphocyte ratio, mortality, NHANES (national health and nutrition examination survey)

## Abstract

**Background:**

To date, no studies have investigated the correlation between the neutrophil-to-lymphocyte ratio (NLR) and the long-term risk of mortality in individuals with both coronary heart disease (CHD) and hypertension. This study aims to evaluate the association between NLR and all-cause and cardiovascular mortality among this patient population.

**Methods:**

National Death Index (NDI) and National Health and Nutrition Examination Survey (NHANES 2001–2018) were the data sources. A nonlinear association between the NLR and mortality risk was shown by restricted cubic spline (RCS) analysis. Using a weighted Cox proportional hazards model, we quantitatively evaluated the effect of NLR on mortality risk.

The capacity of NLR to forecast survival was assessed by evaluating time-dependent receiver operating characteristic (ROC) curves. A mediating influence analysis was conducted to assess the influence of NLR on mortality through eGFR as a mediator.

**Results:**

The study involved a total of 2136 individuals. During the median follow-up interval of 76.0 months, 801 deaths were recorded. The RCS analysis showed NLR and mortality risk to have a nonlinear relationship. Two groups were established based on the participants’ NLR levels: a group with high NLR (NLR > 2.65) and a group with low NLR (NLR < 2.65). After adjusting for potential confounding factors, the Cox proportional hazards model revealed that participants with an increased NLR faced a significantly higher risk of cardiovascular mortality. (HR 1.58, 95% CI 1.33–1.82, *p* < 0.0001) and all-cause mortality (HR 1.46, 95% CI 1.30–1.62, *p* < 0.0001). An analysis of interactions and data stratification corroborated the validity of our findings. eGFR was identified as a partial mediator in the association between NLR and mortality rates, contributing 12.17% and 9.66% of the variance in all-cause and cardiovascular mortality, respectively. The predictive performance for cardiovascular mortality was quantified using ROC curves, with respective AUC values of 0.67, 0.65, and 0.64 for predictions over 3, 5, and 10 years. The AUC values for all-cause mortality were 0.66, 0.64, and 0.63 for the same time frames.

**Conclusion:**

For patients with CHD and hypertension, an elevated NLR serves as an independent prognostic indicator for both all-cause and cardiovascular mortality.

## 1 Introduction

Coronary heart disease (CHD) is a significant concern in cardiology due to its high incidence, disability, and mortality rates (1, 2). According to the American Heart Association (AHA), the number of deaths attributed to heart disease in the United States surpasses 380,000 annually (3). Hypertension, recognized as a substantial factor in arteriosclerosis, frequently coexists with CHD. Compared to those with CHD alone, individuals with both hypertension and CHD confront heightened health risks. Research has shown that hypertension significantly exacerbates the risk of cardiovascular events in patients with CHD, leading to a higher incidence of strokes and cardiac incidents (4, 5). Therefore, the early detection and treatment of danger indicators are essential for individuals managing both CHD and hypertension to lessen the worldwide burden of cardiovascular diseases.

In the field of cardiovascular disease research, biomarkers such as C-reactive protein (CRP), interleukin (IL), and tumor necrosis factor-alpha (TNF-α) are considered to be associated with poor prognosis in patients with hypertension or coronary heart disease (6). These markers may exert their effects by impairing vascular endothelial function and accelerating the process of atherosclerosis (7, 8). However, their application in predicting the prognosis of cardiovascular disease patients does not always meet expectations.

The neutrophil-to-lymphocyte ratio (NLR), a critical biomarker that indicates immune responses and systemic inflammation, has demonstrated its worth in various medical contexts. Its significance in conditions such as metabolic syndrome, cancer, rheumatoid arthritis, and the severity of COVID-19 is widely recognized (9–13). Numerous studies have consistently demonstrated that high levels of NLR are linked to the occurrence and existence of hypertension (14), as well as several cardiovascular diseases (15). Moreover, research has revealed that an increased NLR significantly correlates with increased mortality risks in individuals diagnosed with acute coronary syndrome (16). Nevertheless, more research into the correlation between NLR and cardiovascular and all-cause mortality in CHD and hypertension patients is needed.

This study aims to investigate the relationship between NLR and the risk for mortality from any cause and cardiovascular-related causes in patients who have both CHD and hypertension. The study will encompass a substantial and demographically diverse population sample that accurately represents the entire nation.

## 2 Materials and methods

### 2.1 Study design

The National Health and Nutrition Examination Survey (NHANES), administered by the Centers for Disease Control and Prevention (CDC), is a comprehensive, cross-departmental study to evaluate the American population’s health condition. This assessment is achieved through surveys, health evaluations, nutritional assessments, and laboratory tests. Once adjusted, the survey’s sampling methodology ensures a representative sample that reflects the demographic diversity of the American population. The research protocol received clearance from the National Centre for Health Statistics Ethics Review Board (NCHS) (17).

We collected data from individuals who participated in the survey from 2001 to 2018. Furthermore, we obtained mortality data from the National Death Index (NDI) through the NCHS to monitor participants’ survival and establish a longitudinal cohort for NHANES. Each participant has provided written consent, affirming their understanding and agreement to participate. The study’s methodology has received clearance from the Institutional Review Board, fulfilling the necessary ethical requirements. Consequently, there is no requirement for further consent forms or supplementary ethical evaluations for this research.

### 2.2 Study population

The study included participants diagnosed with both CHD and hypertension, with data collected from 2001 to 2018. CHD information was obtained by asking participants:” Has a doctor or other health professional ever told you that you had coronary heart disease?” Has a doctor or other health professional ever told you that you had angina, also called angina pectoris?” or “Has a doctor or other health professional ever told you that you had a heart attack (also called myocardial infarction)?” If they answered “Yes” to any of the above questions, they were diagnosed with CHD. Blood pressure was measured by certified medical personnel following a 5-minute rest period, taken on three or four occasions at 30-second intervals, and following standardized protocols. If a person satisfied one or more of the following requirements, they were considered hypertensive: (1) Self-reported diagnosis of hypertension, (2) mean diastolic blood pressure of 90 mm Hg or higher, (3) continuous antihypertensive medication, and (4) mean systolic blood pressure of 140 mm Hg or higher (18). The study focused on individuals with comorbid CHD and hypertension, excluding those lacking essential survival data, critical baseline clinical information, or who were under 18 years old. Finally, the research involved 2,136 eligible patients (**Supplementary Figure S1**).

### 2.3 Measurement of NLR

Routine blood tests are a standard procedure for blood analysis. They assess an individual’s health status and detect a variety of pathological conditions. In this study, complete blood count data were acquired through technology provided by Beckman Coulter. The lymphocyte count divided by the absolute neutrophil count yielded the NLR (19).

### 2.4 Mortality outcomes of the study population

We used the NDI, a database managed by the CDC, to compile our mortality statistics. The participant’s follow-up period commenced upon their enrollment in the study and concluded either upon their demise or the final update of the NDI database on December 31, 2019. Cardiovascular mortality included deaths caused by heart diseases (defined by ICD-10 codes I00–I09, I11, I13, I20–I51) and cerebrovascular diseases (defined by ICD-10 codes I60–I69) by the 10th edition of the International Classification of Diseases (20).

### 2.5 Assessment of covariates

A standardized set of covariates was utilized when analyzing mortality rates related to all causes and cardiovascular diseases. Demographic factors included age, sex, race, and education. Race was classified into non-Hispanic white, non-Hispanic black, Mexican, and other racial groups. Education levels were categorized into three tiers: less than high school, high school or General Educational Development (GED), and higher than high school education. Participants medical histories were reviewed for Cancer, heart failure, smoking conditions, and Body Mass Index (BMI). Individuals who were continuously smoking were categorized as current smokers, whereas individuals who had smoked more than 100 cigarettes during their lives but had given up were classified as former smokers (21). The BMI was computed using the following equation: weight (in kilograms) divided by height (in square meters). The resulting BMI values are classified as follows: normal (BMI less than 25.0 kg/m²), overweight (BMI ranging from 25.0 to 30 kg/m²), and obese (BMI of 30.0 kg/m² or above) (22). Laboratory blood parameters assessed included high-density lipoprotein cholesterol (HDL-C), total cholesterol (TC), serum albumin, estimated glomerular filtration rate (eGFR), and serum creatinine (SCR). The eGFR was calculated using the chronic kidney disease epidemiology collaboration group (CKD-EPI) formula. Chronic kidney disease (CKD) is defined as an individual with an eGFR chronic kidney disease in the range of 15 to 59 mL/min/1.73 m² (23).

### 2.6 Statistical analysis

Adhering to the NHANES protocols for analysis and reporting (24), this research considered the complexities of the sampling design and utilized appropriate sample weights. These weights were calculated from the fasting subset of the Mobile Examination Center (MEC) data collected over 14 years by dividing the two-year MEC weight by 9. Using the maximum rank statistics from the ‘maxstat’ package, we identified the threshold values of the NLR to differentiate between participants in the low and high NLR groups (25). Continuous variables were depicted through median values complemented by their interquartile range (IQR) from the 25th to the 75th percentile. Counts and their respective proportions illustrated categorical data. To examine the nonlinear connection between NLR and death rates from cardiovascular disease and all causes in individuals who had both CHD and hypertension, restricted cubic spline (RCS) analysis was used.

The RCS model is more flexible than the traditional linear regression, allowing it to fit the natural distribution of data without the necessity of predefining the function’s form. By establishing nodes in different NLR ranges, RCS permits the capture and depiction of the underlying trends in the relationship between NLR and mortality, which in turn boosts the model’s interpretability and statistical potency. Additionally, the RCS analysis assists in the identification and management of heterogeneity within the data. To ascertain the configuration of the curve and identify potential inflection points, the RCS model was chosen based on its Akaike Information Criterion (AIC) value being the lowest. The model includes four knots.

This patient population’s unique correlations between NLR and risks of death from any cause and cardiovascular disease were investigated using weighted Cox proportional hazards models. The Schoenfeld residuals test was used to evaluate the proportional hazards hypothesis. The models were stratified into three levels to control for possible confounding factors. Covariates, such as age, gender, race, heart failure, and cancer, which were significantly related to survival status, were included in the models based on theoretical and statistical grounds. To address multicollinearity, variables with variance inflation factors above five were excluded before selecting the remaining variables. The final model was demonstrated after accounting for age, gender, race, education level, smoking conditions, BMI, HDL, serum albumin, TC, and eGFR.

The Kaplan-Meier method was applied for survival analysis to evaluate the survival probabilities for individuals diagnosed with CHD and hypertension at different NLR levels, with the log-rank test employed for comparisons. Stratification and interaction analyses were conducted to examine the influence of several factors, including age, gender, smoking conditions, race, cancer, and heart failure. The ‘time ROC’ software was used to assess the prediction value of NLR for survival results at various time intervals (26). To assess whether the eGFR acts as a mediator between the NLR and mortality, we employed a mediation model of path analysis. We first established a direct effect model of NLR on mortality, then included eGFR as a mediating variable in the model, and assessed the impact of NLR on eGFR and the impact of eGFR on mortality separately. Version 4.3.1 of the R statistical package was used to perform the analysis. With two-sided testing, p-values under 0.05 were significant.

## 3 Results

### 3.1 Baseline characteristics

In this research, we enrolled 2,136 individuals with CHD and hypertension, constituting a representative sample of the 7,769,596 patients across the United States (**Supplementary Figure S1**). At 2.65, the optimal NLR threshold was determined, thereby dividing the participants into two categories: one with an NLR exceeding 2.65 (n=753) and another with an NLR at or below 2.65 (n=1,383) (**Supplementary Figure S2**). Upon comparison, discernible disparities were noted between the two groups. The group with elevated NLRs was characterized by an older average age and a predominantly Non–Hispanic White. Additionally, a smaller percentage of this group were non-smokers. Regarding clinical parameters, the group with higher NLRs exhibited reduced TC, serum albumin, lymphocyte count, and eGFR levels compared to those with lower NLRs (**Table 1**).

**Table 1:** Baseline characteristics of included participants.

Variables	Total (n = 2136)	Lower NLR (n = 1383)	Higher NLR (n = 753)	*P* value
Age, years	69.00 (61.00, 77.25)	67.00 (59.00, 76.00)	72.00 (64.00, 80.00)	<0.0001
Sex, %				<0.0001
Male	1293(61.5)	792(57.3)	501(66.5)	
Female	843 (39.5)	591 (42.7)	252 (33.5)	
Ethnicity, %				<0.0001
Non–Hispanic White	1218 (57.0)	715 (51.7)	503 (66.8)	
Mexican American	209 (9.8)	138 (10.0)	71 (9.4)	
Other Race	293 (13.7)	204 (14.8)	89 (11.8)	
Non–Hispanic Black	416 (19.5)	326 (23.6)	90 (12.0)	
Educational level, %				0.2442
Less than high school level	729 (34.1)	487 (35.2)	242 (32.1)	
High school or equivalent	527 (24.7)	328 (23.7)	199 (26.4)	
Greater than high school level	880 (41.2)	568 (41.1)	312 (41.4)	
smoking conditions, %				0.0021
Never smoker	804 (37.6)	552 (39.9)	252 (33.5)	
Former smoker	904 (42.3)	547 (39.6)	357 (47.4)	
Current smoker	428 (20.0)	284 (20.5)	144 (19.1)	
BMI, kg/m^2^	29.69 (26.05, 34.10)	29.70 (26.20, 34.26)	29.65 (25.66, 33.80)	0.2652
BMI Category, %				0.2171
< 25.0	408 (19.1)	250 (18.1)	158 (21.0)	
25.0-29.9	706 (33.1)	469 (33.9)	237 (31.5)	
> 29.9	1022 (47.8)	664 (48.0)	358 (47.5)	
Cancer, %				<0.0001
Yes	477 (22.3)	274 (19.8)	203 (27.0)	
No	1653 (77.4)	1103 (79.8)	550 (73.0)	
Not recorded	6 (0.3)	6 (0.4)	0 (0.0)	
HF, %				<0.0001
Yes	608 (28.5)	350 (25.3)	258 (34.3)	
No	1504 (70.4)	1015 (73.4)	489 (64.9)	
Not recorded	24 (1.1)	18 (1.3)	6 (0.8)	<0.0001
eGFR, mL/min/1.73m^2^	73.51 (56.93, 91.21)	76.79 (60.87, 93.01)	67.99 (49.56, 86.20)	<0.0001
Albumin, g/L	41.00 (39.00, 43.00)	42.00 (40.00, 44.00)	41.00 (39.00, 43.00)	<0.0001
TC, mmol/L	4.50 (3.83, 5.33)	4.58 (3.93, 5.46)	4.32 (3.67, 5.07)	<0.0001
HDL, mmol/L	1.19 (1.01, 1.50)	1.19 (1.01, 1.50)	1.19 (0.98, 1.47)	0.9192
SCR, umol/L	88.40 (73.15, 108.73)	87.52 (70.72, 106.08)	97.24 (79.56, 119.34)	<0.0001
Neutrophil, × 10^9^/L	4.30 (3.30, 5.40)	3.80 (3.00, 4.70)	5.40 (4.40, 6.50)	<0.0001
Lymphocyte, × 109/L	1.90 (1.50, 2.40)	2.20 (1.80, 2.70)	1.40 (1.20, 1.80)	<0.0010

BMI, body mass index; HDL, high-density lipoprotein cholesterol; HF, Heart failure; TC, total cholesterol; SCR, serum creatinine; eGFR, estimated glomerular filtration rate. Continuous variables are presented as the mean and 95% confidence interval, category variables are described as the percentage and 95% confidence interval.

### 3.2 Associations of the NLR with all−cause mortality

**Figure 1:**
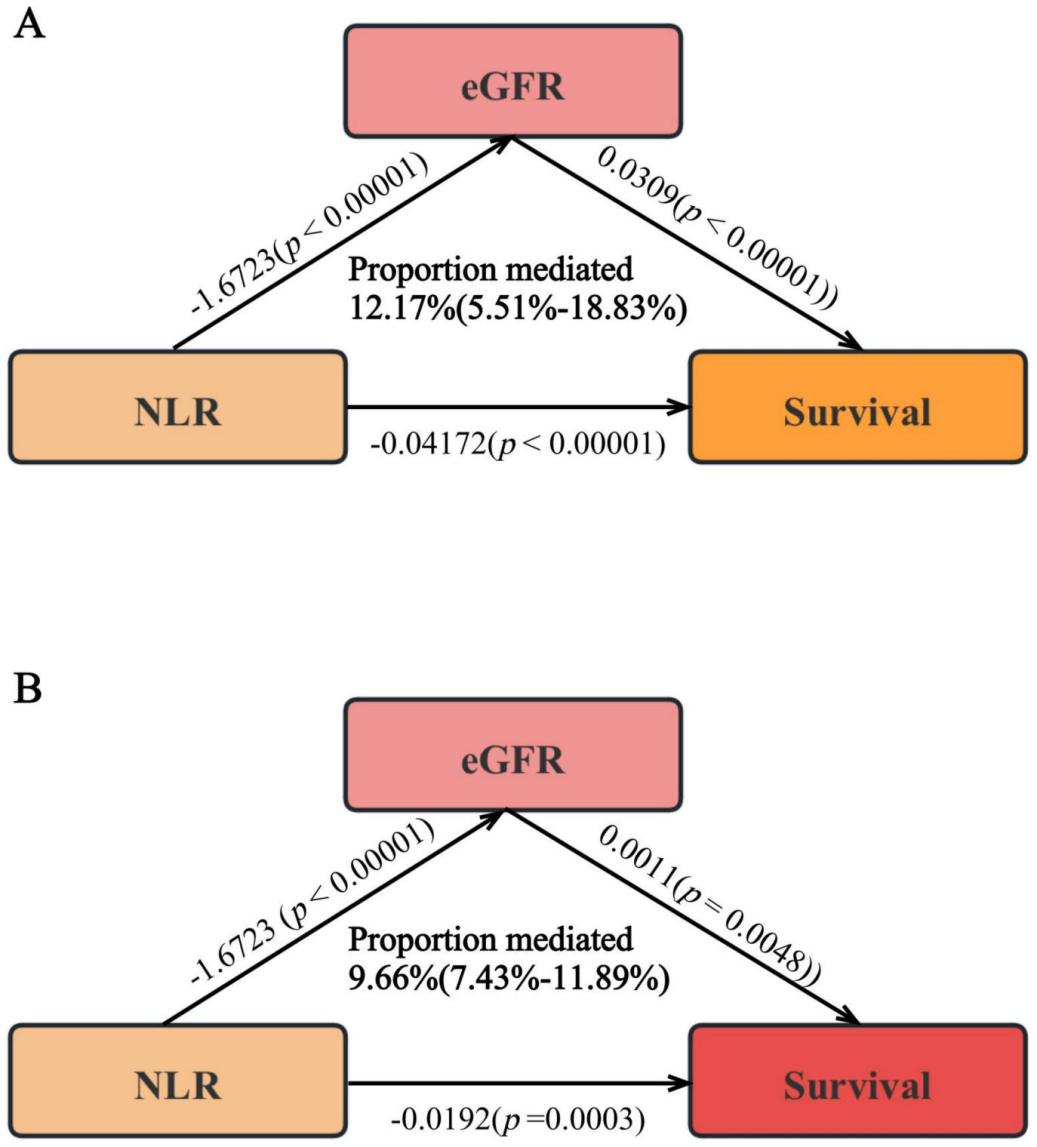
The association of NLR with all-cause **(A)** and cardiovascular mortality **(B)**. Hazard ratios were adjusted for age, sex, race, heart failure, cancer, education, smoking conditions, BMI, HDL, albumin, TC, eGFR.

Throughout the median duration of observation of 76.0 months, which included an interquartile range (IQR) from 39.8 to 85.5 months, among the cohort of 2,136 individuals, 801 (37.4%) fatalities were recorded. Cardiovascular conditions were responsible for 326 (15.2%) of the total deaths. Using RCS analysis, we discerned that the NLR significantly correlated with mortality from all causes in a positive nonlinear trend. (*p* for non-linear = 0.0460) (**Figure 1A**). In the initial analysis (Model 1), an elevated NLR was found to be significantly and positively linked to the risk of death from all causes, with a hazard ratio (HR) of 1.26 (95% confidence interval (CI): 1.22-1.29, *p* < 0.0001) (**Table 2**). This correlation remained after controlling for other factors; in Model 2, the probability of mortality from any cause increased by 20%. (HR: 1.20, 95% CI: 1.16-1.23, *p* < 0.0001) and by 16% in Model 3 (HR: 1.16, 95% CI: 1.13-1.20, *p* < 0.0001) (**Table 2**) for each unit increase in NLR.

**Table 2:** The relationships between NLR and mortality in patients with comorbid CHD and hypertension.

Characteristic	Model 1	*P* value	Model 2	*P* value	Model 3	*P* value
	HR (95% CI)		HR (95% CI)		HR (95% CI)	
All-cause mortality						
NLR	1.26(1.22,1.29)	< 0.0001	1.20(1.16, 1.23)	< 0.0001	1.16(1.13, 1.20)	< 0.0001
NLR category						
Lower NLR	Ref		Ref		Ref	
Higher NLR	2.00(1.84, 2.16)	< 0.0001	1.60(1.45, 1.75)	< 0.0001	1.46(1.30, 1.62)	< 0.0001
Cardiovascular mortality						
NLR	1.27(1.23, 1.31)		1.22(1.17, 1.26)		1.19(1.13, 1.24)	
NLR category						
Lower NLR	Ref		Ref		Ref	
Higher NLR	2.20(2.01, 2.40)	< 0.0001	1.73(1.49, 1.97)	< 0.0001	1.58(1.33, 1.82)	< 0.0001

Model 1 was unadjusted; Model 2 was adjusted for age, sex, race, heart failure, and cancer; Model 3 was adjusted for age, sex, race, heart failure, cancer, education, smoking conditions, BMI, HDL, albumin, TC, eGFR.

**Figure 2:**
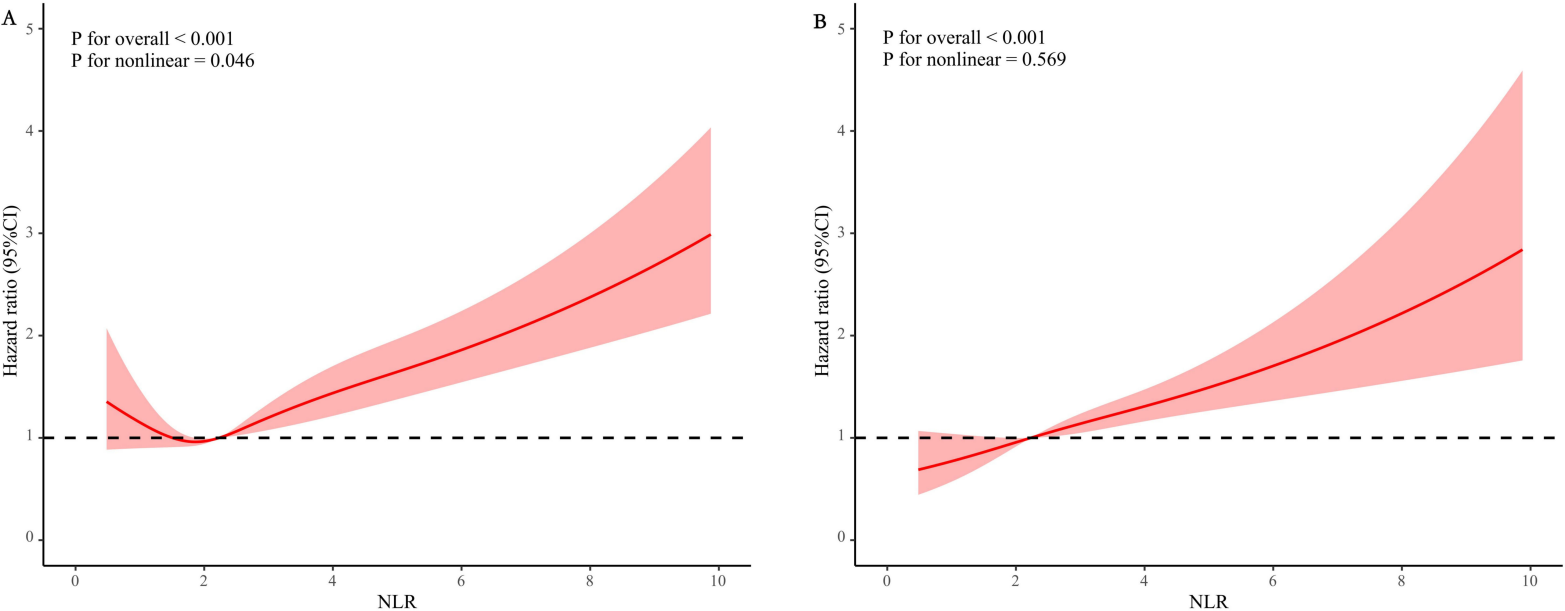
Kaplan-Meier curves of the survival rate with higher (> 3.5) and lower (≤ 3.5) NLR values. **(A)** All-cause mortality; **(B)** cardiovascular mortality.

The survival curves study revealed a significantly lower survival rate for the group with a higher NLR in comparison to the group with a lower NLR (*p* < 0.0001) (**Figure 2A**). The Cox regression analysis illustrated a significantly higher risk of death from all causes for individuals with elevated NLR, as evidenced by the progression from Model 1 (HR: 2.00, 95% CI: 1.84-2.16, *p* < 0.0001) to Model 2 (HR: 1.46, 95% CI: 1.30-1.62, *p* < 0.0001), and Model 3 (HR: 1.46, 95% CI: 1.30-1.62, *p* < 0.0001) (**Table 2**). This trend underscores a substantial rise in death from all causes rates among participants with higher NLR values.

Subgroup analyses, considering factors such as age, sex, race, smoking conditions, cancer, and heart failure, demonstrated consistent correlations across all groups except for the Mexican American populations, where no significant interactions were found between NLR and the assessed characteristics (*p* for interaction > 0.05) (**Table 3**).

**Table 3:** Subgroup analysis of the associations between NLR and mortality.

Characteristics	All−cause mortality			*P* interaction	Cardiovascular mortality			*P* interaction
	Lower NLR	Higher NLR			Lower NLR	Higher NLR		
		HR (95% CI)	*P* value			HR (95% CI)	*P* value	
Age				0.9162				0.4897
<65	Ref	1.73 (1.25, 2.39)	0.0012		Ref	1.47 (0.81, 2.65)	0.2021	
≥65	Ref	1.71 (1.46, 2.00)	<0.0001		Ref	1.87 (1.47, 2.37)	<0.0001	
Sex				0.9512				0.7948
Male	Ref	1.91 (1.60, 2.28)	<0.0001		Ref	2.06 (1.58, 2.69)	<0.0001	
Female	Ref	1.93 (1.53, 2.44)	<0.0001		Ref	1.97 (1.34, 2.90)	0.0011	
Ethnicity				0.5085				0.2257
Non–Hispanic White	Ref	1.79 (1.51, 2.12)	<0.0001		Ref	1.96 (1.51, 2.55)	<0.0001	
Mexican American	Ref	1.49 (0.9, 2.48)	0.1221		Ref	0.94 (0.38, 2.28)	0.8852	
Other Race	Ref	2.15 (1.27, 3.62)	0.0042		Ref	2.98 (1.38, 6.45)	0.0053	
Non–Hispanic Black	Ref	2.27 (1.56, 3.31)	<0.0001		Ref	2.44 (1.36, 4.38)	0.0032	
smoking conditions				0.2944				0.1294
Never	Ref	1.74 (1.37, 2.22)	<0.0001		Ref	1.76 (1.24, 2.49)	0.0020	
Former	Ref	2.18 (1.77, 2.67)	<0.0001		Ref	2.11 (1.53, 2.92)	<0.0001	
Current	Ref	1.77 (1.27, 2.45)	0.0022		Ref	3.51 (1.93, 6.38)	<0.0001	
HF				0.0525				0.0616
Yes	Ref	2.43 (1.90, 3.10)	<0.0001		Ref	2.35 (1.61, 3.42)	<0.0001	
No	Ref	1.72 (1.44, 2.05)	<0.0001		Ref	1.94 (1.48, 2.55)	<0.0001	
Cancer				0.3974				0.4195
Yes	Ref	2.09 (1.60, 2.73)	<0.0001		Ref	2.40 (1.56, 3.70)	<0.0001	
No	Ref	1.84 (1.56, 2.17)	<0.0001		Ref	1.94 (1.51, 2.51)	<0.0001	

HRs were adjusted for age, sex, race, heart failure, cancer, education, smoking conditions, BMI, HDL, albumin, TC, eGFR.

### 3.3 Associations of the NLR with cardiovascular mortality

The RCS analysis showed that the NLR significantly correlated positively with the probability of mortality from cardiovascular causes (*p* for non-linear = 0.5690) (**Figure 1B**).

Increasing NLR values positively correlated with the risk of death from cardiovascular disease in the baseline model (Model 1). (HR:1.27, 95% CI 1.23- 1.31, *p* < 0.0001) (**Table 2**). After rigorous multivariable adjustment (Model 3), the probability of mortality from cardiovascular causes increased by 19% for every unit rise in NLR. (HR:1.19, 95% CI 1.13-1.24, *p* < 0.0001) (**Table 2**).

Survival curve analysis indicated that participants with higher NLR values experienced a significant decrease in survival rates compared to those with lower values (*p* < 0.0001) (**Figure 2B**). The Cox proportional hazards model reinforced these findings, highlighting a significant increase in cardiovascular mortality among individuals with higher NLR values, with HRs of 2.20 (95% CI 2.01-2.40, *p* < 0.0001) in Model 1, 1.73 (95% CI 1.49-1.97, *p* < 0.0001) in Model 2, and 1.58 (95% CI 1.33-1.82, *p* < 0.0001) in Model 3 (**Table 2**).

To investigate the correlation between NLR and rates of death from cardiovascular causes, stratified analyses were performed, taking into consideration factors including age, gender, race, smoking conditions, cancer, and heart failure. Consistent relationships were observed across most strata, except for the Mexican Americans and those aged under 60 years. No significant interactions were found between NLR and the assessed characteristics (*p* for interaction > 0.05) (**Table 3**).

### 3.4 The predictive value of NLR for all-cause and cardiovascular mortality

**Figure 3:**
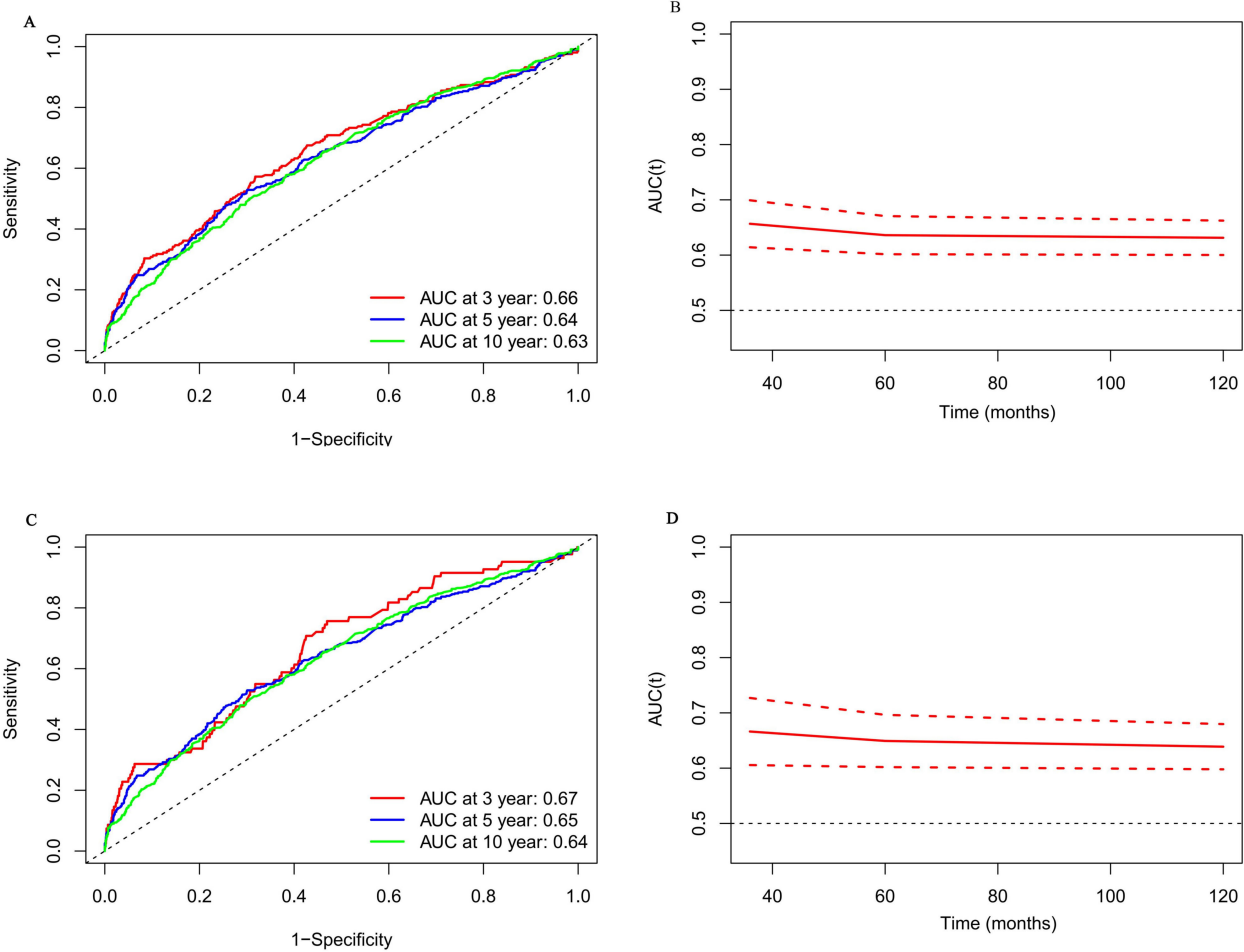
Time-dependent ROC curves and time-dependent AUC values (with 95% confidence band) of the NLR for predicting all-cause mortality **(A, B)** and cardiovascular mortality **(C, D)**.

In the 3-year, 5-year, and 10-year intervals, the NLR had AUC of 0.66, 0.65, and 0.63 for predicting all-cause death, respectively, according to the time-dependent ROC curve analysis (**Figures 3A, B**). Similarly, during the same periods, the NLR had an AUC of 0.67, 0.65, and 0.64 for predicting cardiovascular death (**Figures 3C, D**). These findings suggest that NLR exhibits consistent predictive accuracy for mortality across various time frames. Furthermore, the predictive efficacy of lymphocytes and neutrophils as prognostic indicators in individuals with CHD and hypertension was assessed. The assessment showed that the NLR was more accurate than death projections based on lymphocytes and neutrophils alone over 3,5,10 years (**Supplementary Figure S3**).

### 3.5 Mediation analysis of NLR for all-cause and cardiovascular mortality

**Figure 4:**
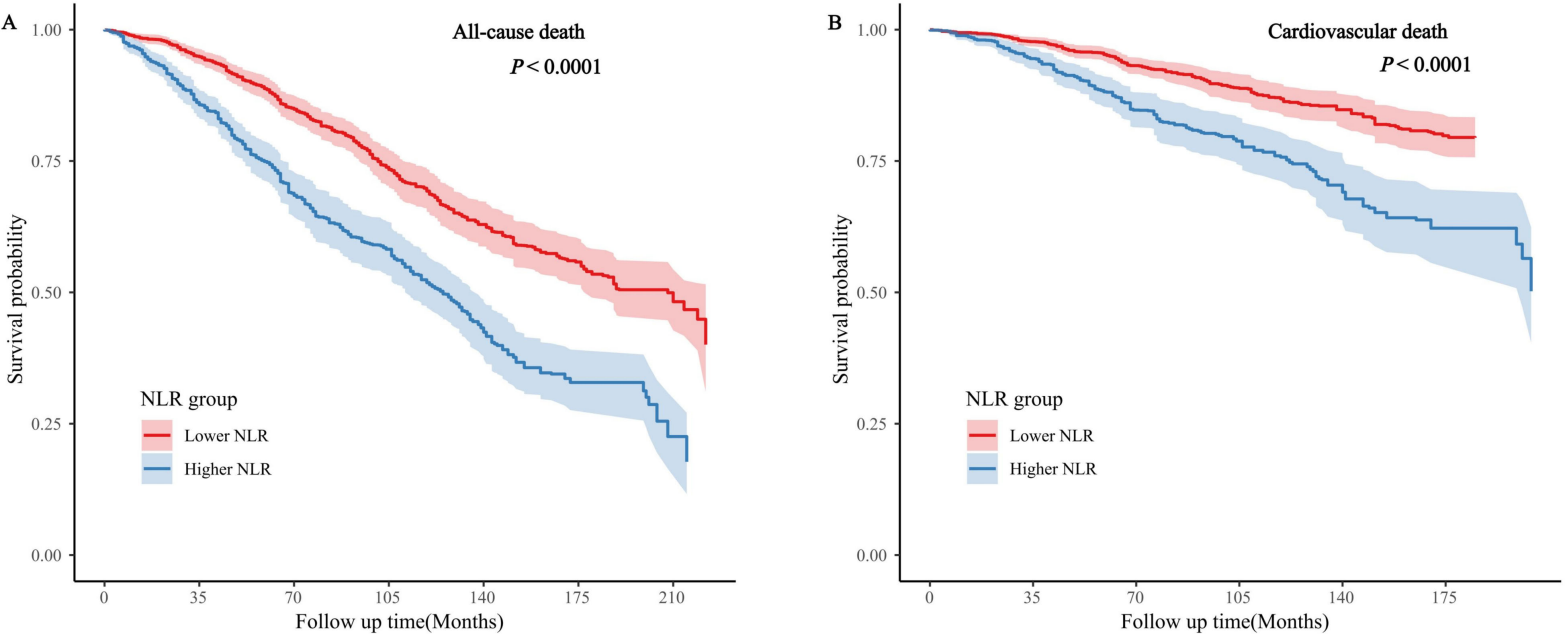
The mediating effect of eGFR on the relationship between NLR and survival [**(A)** all-cause death; **(B)** cardiovascular death]. Adjusted for age, sex, race, heart failure, cancer, education, smoking conditions, BMI, HDL, albumin, TC, eGFR.

This study explored the role of the eGFR as a mediator between the NLR and the dangers of mortality from any cause and cardiovascular disease. The results demonstrated a notable inverse relationship between NLR and eGFR. (β ± SE = -1.6723 ± 0.2879, *p* < 0.00001). Further, cardiovascular disease and all-cause mortality rates were both found to be lower in patients with higher eGFRs (All-cause: β ± SE = 0.0309 ± 0.0051, *p* < 0.00001; Cardiovascular: β ± SE = 0.0011 ± 0.0004, *p* = 0.0048). Consequently, 12.17% of the total mortality was attributable to NLR’s mediating effect on eGFR (95% CI 5.51% - 18.83%), and 9.66% was attributable to its mediating effect on the risk of cardiovascular mortality (95% CI 7.43% - 11.89%) (**Figures 4A, B**).

## 4 Discussion

In our study, we aimed to analyze a cohort of people who had CHD and hypertension to determine whether the NLR was associated with an increased risk of death from cardiovascular disease or any cause. By conducting a comprehensive analysis of health examination data from 2,136 individuals across nine NHANES cycles from 2001 to 2018, we discovered that there was a significant association between elevated NLR levels and an increased risk of mortality from any cause and cardiovascular disease, validating NLR as a strong independent predictor of poor survival outcomes. Importantly, NLR demonstrated superior predictive accuracy compared to isolated assessments of lymphocytes or neutrophils for estimating mortality risks over periods of 3, 5, and more than ten years. Additional mediation analysis revealed that the eGFR mediates the relationship between NLR and death. Our results were robust to sensitivity and stratified analyses.

As an index that integrates two countervailing immune pathways, NLR shows greater predictive power than individual neutrophil or lymphocyte measures (27). New evidence highlights the importance of immunological responses and inflammation in CHD development and progression, with oxidized low-density lipoproteins and inflammatory cells interacting to promote atherosclerotic plaque formation and progression (28). Furthermore, in cardiovascular disorders such as unstable angina, acute coronary syndrome, and ischemic stroke, NLR has validated its role as a mortality risk predictor (29, 30). It suggests the potential of NLR as a pivotal metric for predicting disease progression and assessing fatality risks in various conditions. However, the underlying influence of NLR in patients with CHD and hypertension, along with its nonlinear characteristics, warrants further investigation.

Our research identified significant correlations between high NLR and various characteristics in patients with concomitant CHD and hypertension, including advanced age, Caucasian non-Hispanic race, prevalent smoking habits, heart failure, presence of malignancies, and reduced eGFR (**Table 1**). It appears that the relationship between NLR and the risk of death from all causes varies with various levels of NLR since we found a non-linear correlation between the two using the RCS analytical framework. An elevated NLR may indicate an imbalance in the inflammatory response driven by neutrophils and modulated by lymphocytes, which could exacerbate inflammation and act as a critical factor in atherosclerosis progression. This pathological mechanism not only increases plaque instability but may also lead to plaque rupture and erosion, potentially leading to acute myocardial infarction (AMI) and other severe complications (31–33). Current studies emphasize that patients with elevated NLR levels are at a higher risk of adverse clinical outcomes, with an increased probability of fatality, myocardial infarction, and major adverse cardiovascular and cerebrovascular events (MACCE) (34, 35). These findings suggest a strong connection between elevated NLR and unfavorable prognoses in individuals with CHD and hypertension.

The analysis also indicates that when comparing the groups with high and low NLR, mortality rates due to cardiovascular disease and all causes show statistically significant differences. (**Figure 2** and **Table 2**). Furthermore, considering NLR as a continuous variable reinforces its predictive value for estimating the risks of death from any cause and cardiovascular disease in the subsequent 3, 5, and 10 years (**Figure 3**). While NLR may have limitations when used alone as a predictive marker, it has a broader range of applications. It is more cost-effective than conventional markers like C-reactive protein (CRP) and fibrinogen. This makes it a valuable tool for assessing inflammation and immune status (36), as supported by the research findings of Pourafkari et al. (37). NLR can also help physicians to stratify patients more finely. This stratification can identify patients who may benefit most from specific treatments, thereby achieving more effective resource allocation.

In the stratified analysis of this study, which controlled for confounding factors (**Table 2**), a comparatively poorer overall survival prognosis was observed among patients with concurrent CHD and hypertension who had elevated NLR. However, there was no significant increase in the risk of cardiovascular death among those younger than 60 years old.

A similar lack of a clear trend was observed in the risks of death from any cause or cardiovascular disease mortality among Mexican Americans (**Table 3**). It is essential to emphasize that within the group with higher NLR, the number of deaths among participants under 60 (21 deaths) and Mexican Americans (20 deaths) was too small to draw a definitive conclusion.

During the mediation analysis, it was found that in the group of individuals with comorbid CHD and hypertension, NLR is associated with increased all-cause and cardiovascular mortality rates through the reduction of eGFR, suggesting a possible underlying mechanism (**Figure 4**). A wealth of research consistently supports that low-grade chronic inflammation is prevalent not only in CHD but also in other comorbid conditions. This inflammation is closely related to renal disorders with an inflammatory and immune basis, such as diabetic nephropathy and chronic kidney disease (38, 39). Furthermore, increased NLR is associated with a higher neutrophil count and a lower lymphocyte count, which may lead to immune system dysregulation. Such an inflammatory state could trigger the observed decline in glomerular filtration rate (GFR) in individuals with elevated NLR. This inflammatory state may also lead to endothelial dysfunction and fibrosis, decreasing eGFR (40). The decline in eGFR is indicative not just of worsening renal function but also of a range of pathological processes, including increased inflammation, microvascular disease, and oxidative stress factors that are commonly implicated in the progression of cardiovascular diseases and chronic kidney disease and may ultimately lead to increased all-cause mortality (41).

A principal strength of this study lies in its comprehensive sample inclusion and extensive long-term follow-up, which bolster the reliability of the research outcomes and the validity of the statistical analyses conducted. This study also utilized various statistical methods to control the impact of confounding factors on the research outcomes, thereby making the results more robust. For those with CHD and hypertension, this is the first study that thoroughly assesses the link between the NLR and the long-term risks of death from any cause and cardiovascular causes. Furthermore, sampling weighting procedures have greatly improved how our conclusions may be applied to the broader American population. The study also has limitations. Firstly, although we have taken into account many potential confounding factors, the NHANES data lacks clinical information on some important confounding factors such as sepsis and systemic inflammation, which prevents further analysis and discussion in this study. Future research will need to obtain more detailed clinical data to effectively control these confounding factors. Secondly, our primary focus is on the NLR as an inflammatory marker, excluding other blood cell ratio indicators or standard biochemical indicators that may be associated with cardiovascular diseases. Specifically, we did not compare and evaluate the NLR with the ratio of monocytes to lymphocytes, the ratio of platelets to lymphocytes, the mean platelet volume to platelet count ratio, and indicators such as CRP, troponin I, and creatine phosphokinase-MB (CPK-MB). These indicators may provide an additional perspective on the role of inflammation in cardiovascular diseases, and future studies will consider including these indicators. Finally, the study relies on self-reported diagnoses of CHD and hypertension from participants. Although several strategies were employed to reduce systematic biases during data collection, the possibility of reporting bias cannot be entirely ruled out (42). Future studies adopting more objective clinical data collection methods may help to mitigate this limitation.

## 5 Conclusion

In summary, elevated NLR is significantly and independently associated with the risk of all-cause and cardiovascular mortality in patients with CHD combined with hypertension. Notably, due to its various advantages, NLR can serve as a high-quality candidate prognostic marker. Additionally, eGFR appears as a mediating factor in the relationship between NLR and mortality. These findings suggest that the use of NLR in clinical practice can greatly enhance the precision of patient stratification. By identifying patients who may benefit most from specific treatments, NLR can assist physicians in tailoring more personalized treatment plans for each patient, leading to more effective allocation of medical resources. However, we also recognize the need for further research to confirm the clinical application value of NLR and to explore its combined effects with other biomarkers. Future studies will focus on determining the optimal thresholds for NLR in cardiovascular disease management and how to integrate it into existing risk assessment tools.

## Data Availability

The original contributions presented in the study are included in the article/**Supplementary Material**. Further inquiries can be directed to the corresponding author.
