# Supplementary material for: Neutrophil-to-lymphocyte ratio as a predictor of all-cause and cardiovascular mortality in coronary heart disease and hypertensive patients: a retrospective cohort study
Source: Front Endocrinol (Lausanne). 2024 Aug 21;15:1442165. doi: 10.3389/fendo.2024.1442165 (PMC11371692; doi:10.3389/fendo.2024.1442165)
Supplement: Supplementary file 1 [file DataSheet1.docx]

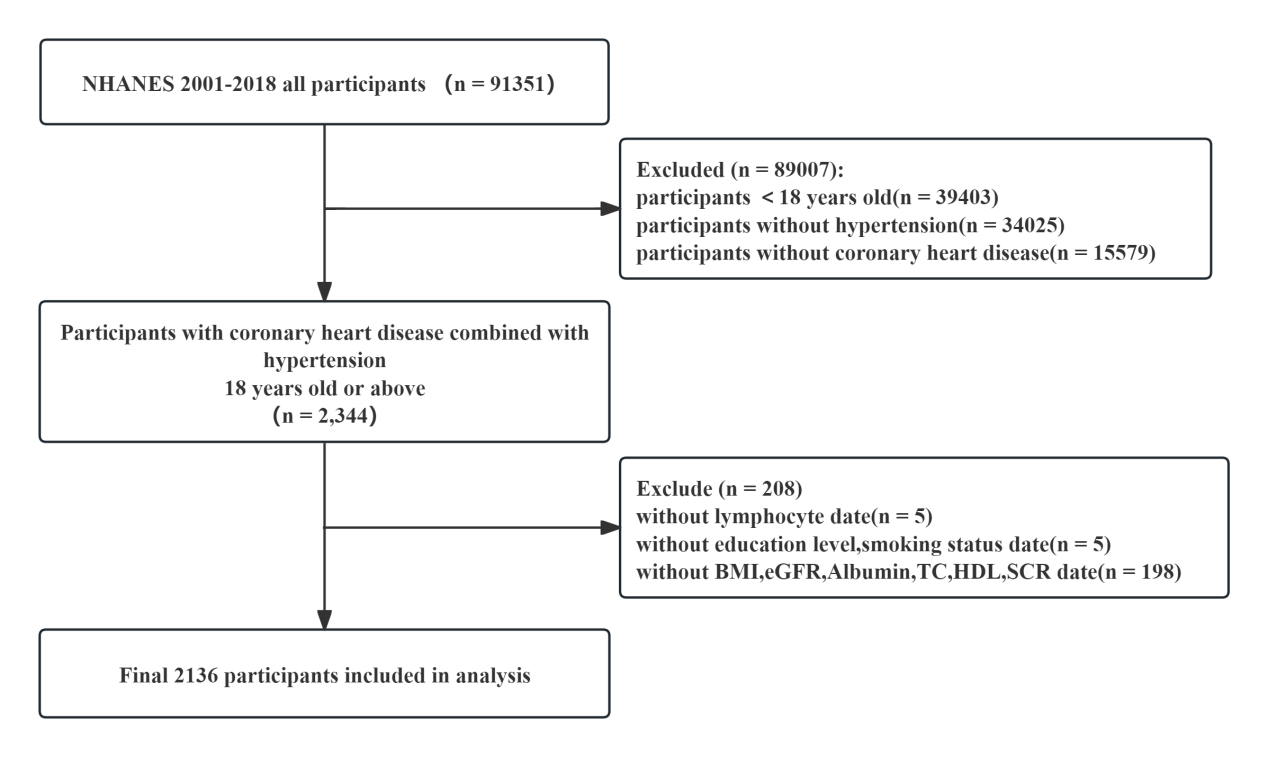


Figure S1. The flow chart of participants in the current study


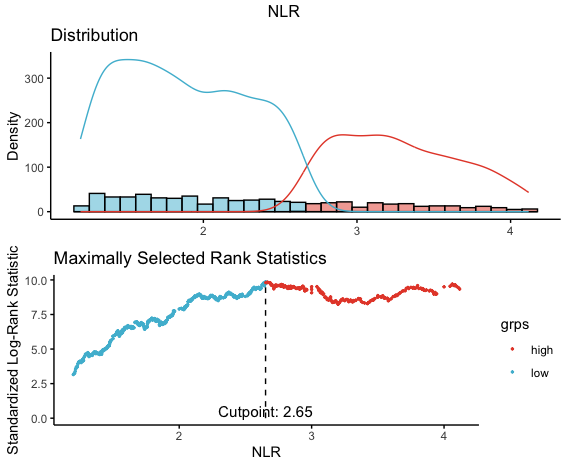


Figure S2. The cutoff point was calculated using the maximally selected rank statistics based on the ‘maxstat’ package


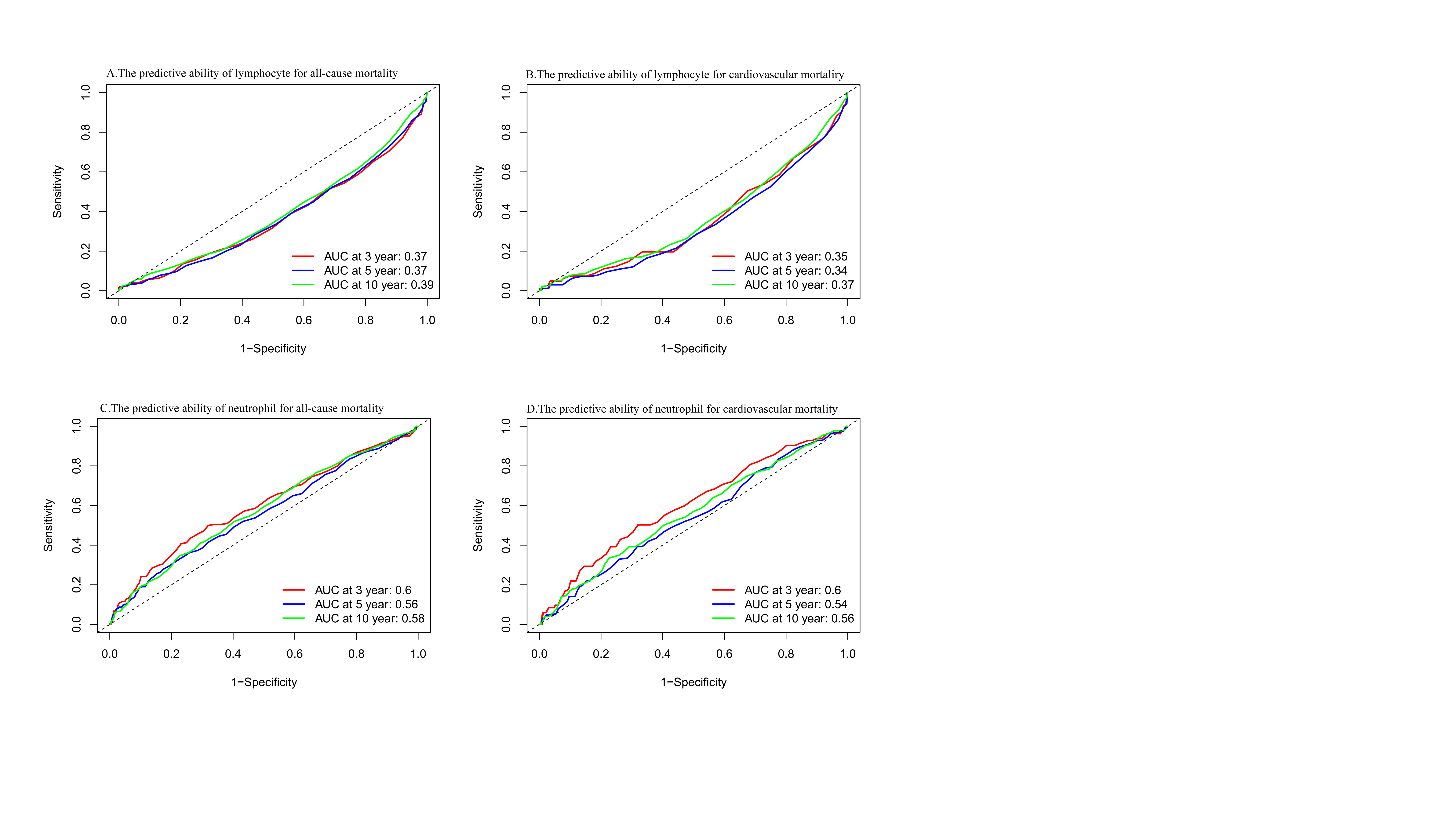


Figure S3. The predictive ability of neutrophil and lymphocyte alone for all‑cause and cardiovascular mortality
